# A Case Report of Probable Dementia With Lewy Bodies Diagnosed Through Repeated Episodes of Depressed Consciousness

**DOI:** 10.7759/cureus.67581

**Published:** 2024-08-23

**Authors:** Kazuki Miyaue, Hiroki Isono

**Affiliations:** 1 Department of General Medicine, HITO Medical Center, Ehime, JPN

**Keywords:** emergency department, primary care, differential diagnosis, altered mental status, dementia with lewy bodies

## Abstract

This case report details the diagnostic challenges of an 84-year-old male with unrecognized dementia with Lewy bodies (DLB) who presented to the emergency department (ED) with episodes of unresponsiveness and depressed consciousness. Despite normal initial laboratory and imaging tests, recurrent symptoms prompted further evaluation, which, along with a detailed history and physical examination, led to the DLB diagnosis. This case underscores the importance of considering DLB in patients with unexplained recurrent depressed consciousness and highlights the importance of clarifying the etiologies of dementia.

## Introduction

Dementia with Lewy bodies (DLB) is the second most common neurodegenerative dementia and is characterized by fluctuating cognition, visual hallucinations, Parkinsonism, and rapid eye movement (REM) sleep behavior disorder [1]. It is sometimes difficult to diagnose DLB, leading to misdiagnosis or overlooking DLB [2].

Altered mental status (AMS), including depressed consciousness, is a common chief complaint among older patients in emergency departments (EDs). Various etiologies can cause AMS, and urgent and accurate management is necessary because AMS can be life-threatening [3].

Here, we present a case of DLB with recurrent depressed consciousness in the ED, which is an uncommon and intriguing clinical manifestation that expands our understanding of this complex neurodegenerative disorder.

## Case presentation

An 84-year-old male with a medical history of dementia (with no known etiology), chronic constipation, and benign prostatic hyperplasia presented to the ED after an episode of unresponsiveness. This presentation was particularly concerning as the patient failed to respond to family attempts at interaction on the morning of admission. His medications included silodosin and sennosides. Upon evaluation, his consciousness level was significantly compromised, as evidenced by a Glasgow Coma Scale score of 6 (E1V1M4). His vital signs were within normal limits. Notably, the patient exhibited resistance to eye-opening and a positive arm-drop test. Other neurological examinations did not contribute to our findings.

**Figure 1:**
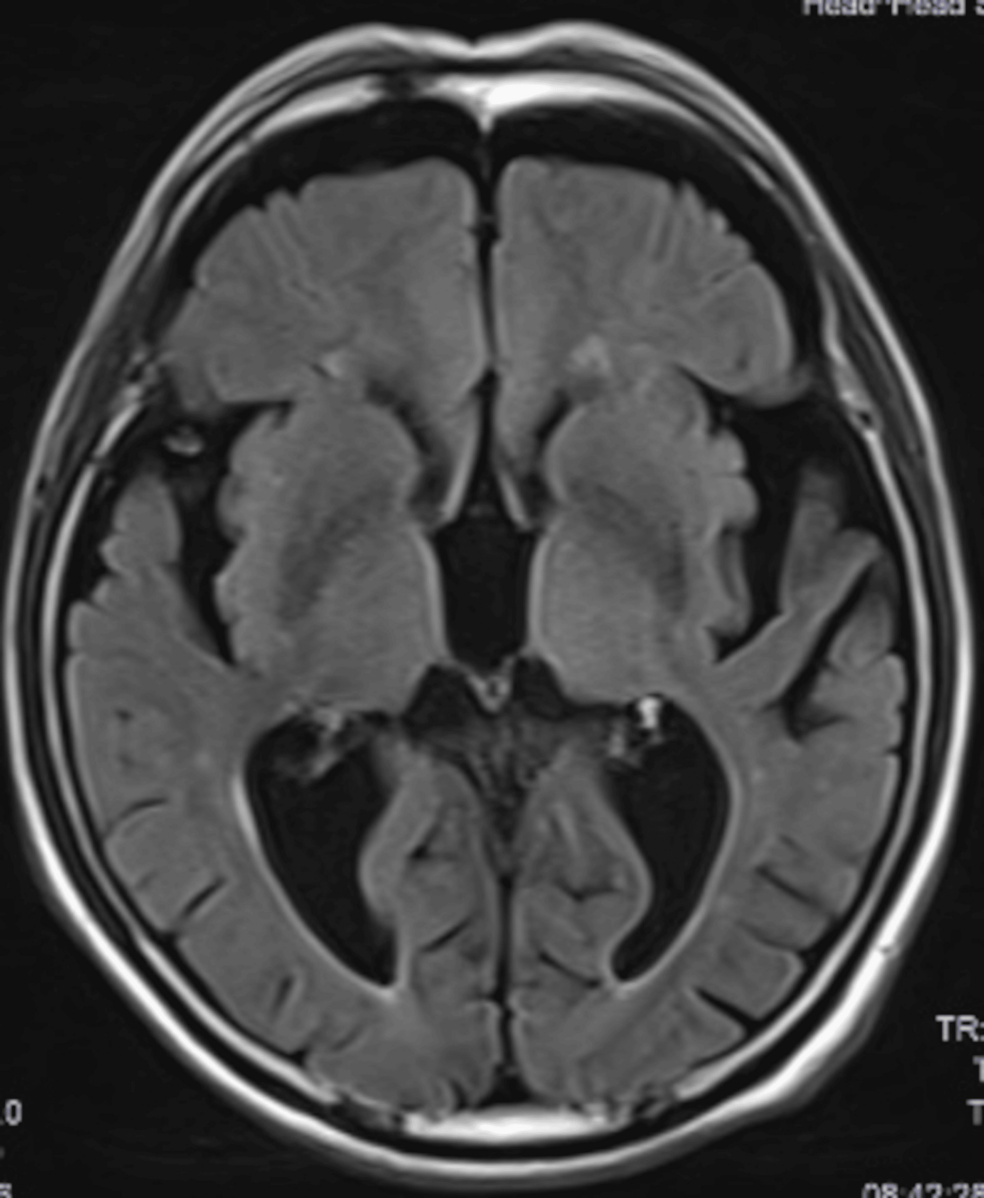
MRI of the brain showing generalized atrophy and an enlargement of the third ventricle MRI: Magnetic resonance imaging

Laboratory investigations, including blood gas analysis and a comprehensive blood panel, revealed no abnormalities that could explain the AMS. Neuroimaging studies, including computed tomography (CT) and magnetic resonance imaging (MRI) of the head, showed generalized atrophy and an enlargement of the third ventricle of the brain (Figure 1), but no other pathological findings to account for the patient’s state.

The patient was admitted for observation and, intriguingly, exhibited a spontaneous return to consciousness within a few hours, regaining the ability to eat. This transient improvement facilitated the discharge on the following day. However, the respite proved fleeting; the patient lost consciousness the following morning and was promptly readmitted. His consciousness level upon rehospitalization mirrored his initial presentation.

During his second hospital stay, additional diagnostic efforts, including electroencephalography and spinal fluid analysis, failed to yield conclusive results. Detailed neurological examination revealed bilateral upper limb muscle rigidity. The clinical diagnosis of probable DLB was made based on this finding, along with a revised medical history that uncovered prior episodes of visual hallucinations and signs of REM sleep behavior disorder, such as shouting, which were associated with dreams that began approximately five years prior to the current presentation.

The patient's hospital course was marked by fluctuating states of consciousness and inadequate nutritional intake, which complicated management. Despite dedicated care, his condition progressively deteriorated, culminating in his passing on the 46th day of hospitalization. 

## Discussion

In the present case, two pivotal findings emerged, underscoring the intricacies and diagnostic challenges of DLB. First, this case highlights the importance of considering DLB as a differential diagnosis in patients presenting with depressed consciousness in the ED, especially when conventional investigations fail to reveal the apparent causes. This scenario is frequently encountered in clinical practice and poses a substantial challenge, demanding a high index of suspicion and thorough evaluation to avoid overlooking DLB, a condition that, while not uncommon, can often masquerade as other disorders owing to its atypical presentation. Second, this case highlights the critical necessity of elucidating the etiology of dementia. The complexity and overlapping symptomatology of various forms of dementia, including DLB, often lead to diagnostic ambiguities, making precise etiological identification important. 

Our initial finding highlights the significance of considering DLB in patients with unexplained depressed consciousness in the ED. The frequency and intensity of cognitive fluctuations in DLB differ not only among individuals but also within the same person [4]. These fluctuations are characterized by rapid changes (lasting from minutes to hours) and more gradual shifts (occurring weekly or monthly) [4]. One study reported that a comatose-like sleep of more than one day in patients with DLB might be a manifestation of fluctuating cognition [5]. Another study indicated that patients with acute-onset AMS presenting to the ED, whose diagnosis was not made in an initial workup, might have undiagnosed DLB [6]. In our case, emergent causes such as stroke, encephalitis, and seizures were initially suspected; however, a thorough investigation of the patient’s medical history and physical examination revealed the underlying cause. Although dopamine transporter single-photon emission computed tomography (DAT-SPECT) and iodine meta-iodobenzylguanidine (MIBG) myocardial scintigraphy were not performed and other possibilities were not completely ruled out, we made the diagnosis of probable DLB based on the diagnostic criteria [7]. Given the presence of the enlargement of the third ventricle, progressive supranuclear palsy is a possibility, but less likely because the patient had cognitive fluctuations, visual hallucinations, and signs of REM sleep behavior disorder, which are specific for DLB. DLB should be considered in older patients with recurrent AMS after ruling out common and life-threatening causes, particularly in those with dementia of unknown etiology.

The identification of specific etiologies of dementia, as exemplified in our case, cannot be overstated. In this case, the primary care physician diagnosed the patient with dementia two years prior; however, the etiology was not investigated. In the assessment of dementia, it is essential to consider degenerative dementias other than Alzheimer's, including DLB, and rule out potentially reversible causes [8]. Early diagnosis of DLB is essential to prevent potentially lethal reactions to inappropriate medications such as antipsychotics and to enable the effective use of beneficial treatments such as cholinesterase inhibitors, which allow for tailored care that improves the patient’s quality of life and optimizes healthcare utilization [9]. Primary care providers (PCPs) have many opportunities to manage patients with dementia; however, DLB is often underdiagnosed in primary care [2], meaning it is essential for PCPs to be more aware of DLB. 

## Conclusions

This case report highlights the importance of considering DLB in older patients with recurrent, unexplained episodes of depressed consciousness in the ED and identifying the underlying etiologies of dementia in primary care.
